# Repurposing Cardiac Glycosides to Potentiate CD47 Blockade through Calreticulin‐mediated Phagocytic Effects for Lung Cancer Treatment

**DOI:** 10.1002/advs.202508245

**Published:** 2025-09-23

**Authors:** Zi‐Han Ye, Wei‐Bang Yu, Mu‐Yang Huang, Yan‐Yan Chen, Le‐Le Zhang, Chung‐Hang Leung, Xiao‐Lei Zhang, Zhenghai Tang, Ting Li, Jin‐Jian Lu

**Affiliations:** ^1^ State Key Laboratory of Quality Research in Chinese Medicine Institute of Chinese Medical Sciences University of Macau Macao 999078 China; ^2^ School of Basic Medical Sciences Chengdu University Chengdu 610106 China; ^3^ National‐Local Joint Engineering Laboratory of Druggability and New Drug Evaluation Guangdong Key Laboratory of Chiral Molecule and Drug Discovery School of Pharmaceutical Sciences Sun Yat‐sen University Guangzhou 510006 China; ^4^ Department of Biomedical Sciences Faculty of Health Sciences University of Macau Macao 999078 China; ^5^ Department of Pharmaceutical Sciences Faculty of Health Sciences University of Macau Macao 999078 China; ^6^ MoE Frontiers Science Center for Precision Oncology University of Macau Macao 999078 China

**Keywords:** calreticulin, cardiac glycosides, CD47 blockade, lung cancer, phagocytosis

## Abstract

The abundance of macrophages within the tumor microenvironment (TME) of lung cancer represents a noteworthy therapeutic target. Exploiting the phagocytic function of macrophages by blocking the “don't eat me” signal, CD47, has shown significant therapeutic potential. However, novel CD47‐targeted combination strategies warrant further investigation. Through an analysis of data obtained from a screening model focused on the macrophage‐mediated killing effect, two cardiac glycosides (CGs), ouabain and digoxin, are shown to increase the capacity of macrophages to kill cancer cells after combination with CD47 antibody. Compared with the control, the combination strategy reduced the tumor volume in different lung cancer models and increased the macrophage phagocytosis rate ≈5‐fold. Mechanistically, in addition to Fc‐FcγR interaction, CGs enhanced the expression of a pro‐phagocytotic signal, calreticulin (CRT). Moreover, PERK inhibitor, ER‐Golgi protein trafficking inhibitor, and siRNA‐mediated knockdown of exocytosis protein exo70, abrogated both CGs‐induced CRT upregulation and the ensuing enhancement of phagocytosis. These findings indicate that CGs drive CRT translocation originates from ER to Golgi apparatus, where it subsequently anchors to the cell surface via exo70‐mediated exocytosis. Overall, this study offers compelling evidence that supports the clinical translation of an innovative combination regimen for the treatment of patients with lung cancer.

## Graphical abstract

Repurposing cardiac glycosides (CGs) significantly strengthens the efficacy of anti‐CD47 antibody for lung cancer treatment by boosting macrophage phagocytosis. This novel combination blocks the “don't eat me” signal and increases dual “eat‐me” signals, Fc‐FcγR and cell surface expression of calreticulin (ecto‐CRT). Mechanistically, CGs drive ecto‐CRT via PERK activation, ER‐Golgi trafficking, and Exo70‐mediated exocytosis, offering new insights for potent anti‐tumor immunotherapy.

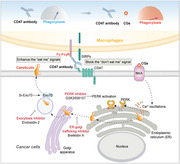

## 1 Introduction

Lung cancer, recognized as one of the largest contributors to cancer diagnosis and cancer‐related deaths worldwide, is categorized into non‐small cell lung cancer (NSCLC, accounting for 80% of all lung cancer cases) and small‐cell lung cancer (SCLC).^[^ ^1^ ^]^ Recent statistics estimated ≈2.27 million new lung cancer cases and 1.25 million deaths in 2025.^2^ In clinical treatment, targeted therapies and immunotherapies have significantly improved outcomes in patients with lung cancer.^[^ ^3^, ^4^ ^]^ For example, targeted therapies, such as tyrosine kinase inhibitors (TKIs), are designed to selectively inhibit specific oncogenic genetic alterations, such as mutations in epidermal growth factor receptor (EGFR) and anaplastic lymphoma kinase (ALK), which are pivotal drivers of tumorigenesis. Osimertinib, a third‐generation EGFR TKI, has exhibited superior therapeutic outcomes in the management of EGFR‐mutant NSCLC, extending the overall survival of patients from less than 12 months to 3 years or more.^[^ ^5^, ^6^ ^]^ In parallel, immune checkpoint inhibitors (ICIs), including pembrolizumab and nivolumab, have also yielded significant clinical benefits in the treatment of advanced NSCLC, leading to durable tumor responses and increased survival rates.^[^ ^7^, ^8^ ^]^ Despite the advancements in these therapeutic modalities, there is considerable room for improvement. Resistance to targeted therapies is an inevitable challenge and limits their long‐term efficacy.^[^ ^9^ ^]^ Additionally, ICIs that primarily target the PD‐L1/PD‐1 axis exhibit relatively low response rates, ≈20%, which represents a significant barrier to optimizing therapeutic outcomes.^[^ ^10^ ^]^ Therefore, the development of novel treatment strategies remains essential for future advancements in anti‐lung cancer therapy.

In the tumor microenvironment (TME), in addition to T cells, there are also various innate immune cells.^[^ ^11^ ^]^ For example, macrophages are one of the largest populations among all types of immune cells in the solid tumor microenvironment.^[^ ^12^ ^]^ These cells play a crucial role in tumor progression and immune regulation, and their complex interaction mechanisms have increasingly emerged as a focal point of research.^[^ ^13^ ^]^ Most of these macrophages play an immunosuppressive role, assisting cancer cells in evading attacks from the immune system.^[^ ^14^ ^]^ The current challenge is how to repurpose these cells to target and kill cancer cells. Macrophage‐mediated phagocytosis could be a pivotal breakthrough, but there are anti‐phagocytic signaling axes in the TME that prevent macrophage phagocytosis.^[^ ^15^ ^]^

CD47, a five‐span transmembrane protein ubiquitously expressed in various cell types, is highly expressed in many types of cancer.^[^ ^16^, ^17^ ^]^ As the most widely studied “anti‐phagocytosis” signal,^[^ ^18^ ^]^ CD47 binds to signal‐regulatory protein alpha (SIRPα) on the surface of myeloid cells, such as macrophages, thereby activating the immunoreceptor tyrosine‐based inhibitory motif intracellularly, which enables cancer cells to evade immune surveillance.^[^ ^17^ ^]^ Previous studies summarized research on agents that target CD47 and revealed that most clinical trials involving CD47‐targeting agents have adopted combination regimens.^[^ ^19^ ^]^ Blocking the CD47‐SIRPα axis alone is currently believed to be insufficient for fully unleashing the phagocytic potential of macrophages, and additional pro‐phagocytic signals are required.^[^ ^15^, ^20^ ^]^ Furthermore, the high level of macrophage infiltration in lung cancer has prompted the investigation of combination strategies involving CD47‐targeting agents in the treatment of lung cancer,^[^ ^21^ ^]^ including combinations with radiotherapy, autophagy inhibitors, and anti‐angiogenic therapies.^[^ ^22^, ^23^, ^24^ ^]^ Our previous research, along with that of others, has demonstrated that the combination of CD47 antibody and EGFR‐TKIs significantly enhances anti‐lung cancer efficacy.^[^ ^25^, ^26^, ^27^ ^]^

An evaluation of macrophage phagocytosis over a short period and residual cancer cells after a prolonged period revealed that CGs such as ouabain and digoxin significantly enhanced the macrophage killing effect mediated by CD47 antibody. Studies have recently focused on exploring the anti‐cancer properties and underlying mechanisms of CGs, diverging from their traditional application in the treatment of heart failure.^[^ ^28^, ^29^, ^30^ ^]^ CGs have been reported to exert anti‐cancer effects by inducing cell death, arresting the cell cycle, inhibiting cell proliferation‐related pathways, and preventing cell migration and invasion.^[^ ^31^, ^32^ ^]^ Notably, ouabain and digoxin were found to be the strongest disruptors of circulating cancer cells and inhibit breast cancer metastasis.^[^ ^33^, ^34^ ^]^ Furthermore, they specifically target senescent cells caused by chemotherapeutic drugs to resensitize them to chemotherapy as senolytic compounds.^[^ ^35^, ^36^ ^]^

This study unraveled that ouabain and digoxin significantly increased the phagocytic activity of macrophages and the efficacy of the CD47 antibody in lung cancer elimination in both in vitro and in vivo models. The observed mechanism involved the Fc‐FcγR interaction mediated by the CD47 antibody, alongside the upregulation of cell surface expression of the “eat me” signal CRT induced by ouabain or digoxin. Furthermore, the detailed translocation path of CRT induced by these two CGs was elucidated.

## 2 Results

### 2.1 CGs Strengthened the Macrophage Killing Effect of CD47 Antibody

**Figure 1:**
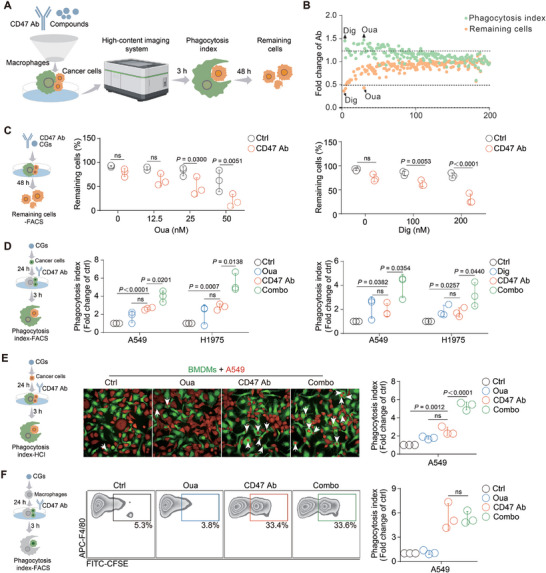
Ouabain and digoxin were identified as compounds potentiating the macrophage killing effect of CD47 antibody. A) A schematic presenting the macrophage phagocytosis‐based high‐content screening process. B) Ouabain and digoxin were identified as the potential compounds to enhance the phagocytosis index and decrease the remaining cells of CD47 antibody, each green spot represented phagocytosis index (fold of CD47 antibody), and each orange spot represented remaining cells (fold of CD47 antibody) of individual compounds. C) Different concentrations of ouabain or digoxin and CD47 antibody were added into the co‐culture model for 48 h, the FACS results indicated that 50 nM ouabain or 200 nM digoxin further decreased the remaining cells after combination with CD47 antibody. (n = 3). D) Macrophages were pretreated with 50 nM ouabain or 200 nM digoxin for 24 h, cancer cells A549 or H1975 with CD47 Ab were added to the macrophage for 3 h, the phagocytosis index was detected by flow cytometry. (n = 3). E) Pretreatment A549 cells with 50nM ouabain for 24h and then add it with or without CD47 Ab to macrophage for 3h, the results were presented by HCI, the white arrow refers the cancer cells underwent phagocytosed by macrophages. (n = 3). F) Pretreatment macrophages for 24 h with 50 nM ouabain and then add cancer cells with or without CD47 antibody to the macrophage for 3 h, the phagocytosis index was detected by flow cytometry. (n = 3). ns, no significance. CD47 Ab: CD47 antibody.

To make our screening results more reliable, the evaluation of the macrophage‐killing effect in the model included not only the calculation of the macrophage phagocytosis index after 3 h but also a co‐analysis of the remaining cancer cells after 48 h (**Figure** **1**A). Both cancer cells undergoing phagocytosis and the remaining cancer cells were evaluated using the HCI algorithm. Six compound plates from the FDA‐approved small compound library were screened by the established model. Two compounds classified as CGs, ouabain and digoxin, exhibited combinational potential after adding simultaneously with CD47 antibody (Figure 1B). Specifically, ouabain combined with CD47 antibody had the best phagocytosis index (1.46‐fold change compared with CD47 antibody alone), whereas digoxin had the best clearance effect (0.39‐fold change compared with CD47 antibody alone) on the remaining cancer cells (Figure 1B). To further confirm the screening results, different concentrations of ouabain or digoxin were combined with CD47 antibody. Both CGs decreased the number of remaining cells at the nanomolar level, and the percentage of remaining cancer cells decreased dramatically when 50 nM ouabain or 200 nM digoxin was combined with CD47 antibody (Figure 1C; Figure S1A, Supporting Information). Notably, the cytotoxic effects of the CGs at these concentrations were modest to lung cancer cells (Figure S1B, Supporting Information). To identify the target cells of two CGs, cancer cells were pretreated with ouabain (50 nM) or digoxin (200 nM) for 24 h, after which the pretreated cells were added to the plates containing macrophages. Consistent with the screening results, ouabain and digoxin further increased the phagocytosis index of CD47 antibody ≈2‐fold, regardless of the FACS results or HCI results (Figure 1D,E; Figure S1A, Supporting Information). In contrast, the phagocytosis index did not change when pretreated CG with macrophages for 24 h and added cancer cells to the co‐culture system (Figure 1F). These results indicate that CGs, including digoxin and ouabain, are the potential enhancers of the killing effect of CD47 blockade, and that the target cells of the CGs in this system might be cancer cells rather than macrophages.

### 2.2 CGs Promoted the Anti‐Cancer Effect of CD47 Antibody via Increased Phagocytosis by TAMs

**Figure 2:**
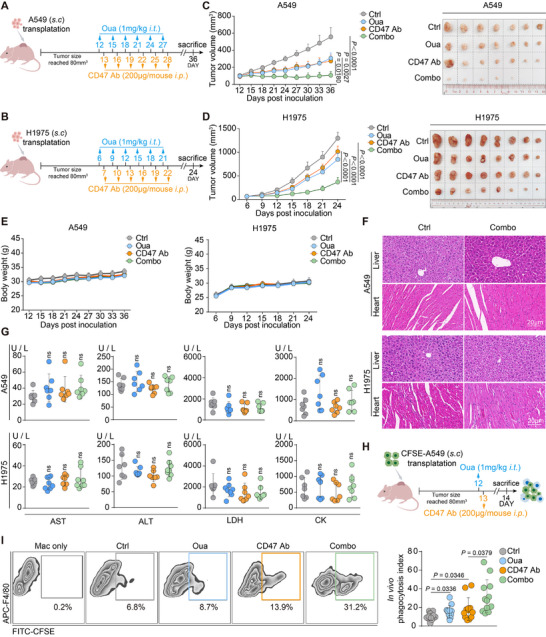
CG enhanced the anti‐tumor effect of CD47 antibody by promoting the phagocytosis of TAMs. A,B) A schematic presenting the administration design of the animal experiments. C,D) Tumor growth curves and a tumor representative image of both A549‐ and H1975‐bearing nude mice models. Data are mean ± s.e.m. of n  =  15 mice per arm for tumor growth in C) and n = 8 mice per arm for tumor growth in (D). E) Body weight change of A549‐ and H1975‐bearing nude mice in the treatment period. F) The H&E staining of the liver and heart of ctrl and combo groups. G) The serum ALT, AST, LDH and CK of two mice models in different treatment groups. H) A schematic presenting the administration design of the in vivo phagocytosis assay and the gating strategy for the in vivo phagocytosis assay. I) The FACS results of in vivo phagocytosis assay, one mouse in each group was selected to represent the in vivo phagocytosis rate, n = 12. Mac only group here was used as negative control for the gating strategy. ns, no significance.

The in vivo therapeutic effect of this newly discovered combination regimen was detected next. A549 and H1975‐bearing BALB/c nude mouse models were used to test the anti‐cancer effect of ouabain co‐administration with antibody targeting CD47 (**Figure** **2**A,B). Treatment with ouabain or CD47 Ab alone affected tumor progression, whereas the combination treatment (combo) significantly shrank the tumor volume in both the A549‐bearing mouse model (Ctrl: 557.2 ± 108.1 mm^3^ vs. combo: 106.7 ± 42.24 mm^3^) and the H1975‐bearing mouse model (Ctrl: 1299 ± 123.1 mm^3^ vs. combo: 371 ± 95.58 mm^3^) (Figure 2C,D). Compared with monotherapy (either ouabain or CD47 Ab), the combo treatment further minimized the tumor volume. In the A549‐bearing mouse model, 5/15 mice even exhibited complete tumor regression, suggesting that these two agents combined had potent anticancer efficacy (Figure 2C). Moreover, neither the single treatment group nor the combo group had no impact on body weight, and there was no histological difference between ctrl and combo groups in either of the two models on the basis of the microanatomy of the liver and heart, and the serum ALT / AST, CK and LDH levels, which were not significantly different among the four groups in the two models (Figure 2E–G), suggesting that this strategy might not cause liver or heart damage.

Importantly, on the basis of the anti‐cancer effect of the combo treatment in mouse models, investigating whether this effect is associated with phagocytosis by TAMs was of interest. An in vivo phagocytosis assay was designed, and after one‐time drug administration,^[^ ^37^ ^]^ the tumors were harvested for further FACS analysis (Figure 2H; Figure S2A,B, Supporting Information). The results confirmed that combo treatment augmented the in vivo phagocytosis by TAMs (Figure 2I). Moreover, to obtain a more comprehensive understanding of the role of macrophages in the combo strategy, we also investigated the immunomodulatory effects on macrophages by CGs. The in vitro macrophage polarization assay indicated that CG did not reprogrammed IL‐4‐stimualted M2‐like macrophage to M1 (Figure S2C, Supporting Information). Animal studies also revealed that CG had no impact in macrophage infiltration and polarization^[^ ^38^ ^]^ (Figure S2D,E, Supporting Information). All the aforementioned results indicated that CG promoted the anti‐cancer effect in different lung cancer‐bearing mouse models by enhancing the phagocytotic functions of TAMs.

### 2.3 Antibody‐Dependent Cellular Phagocytosis (ADCP) Induced by the Combo Strategy Partially Contributed to the Enhanced Phagocytosis of Macrophages

**Figure 3:**
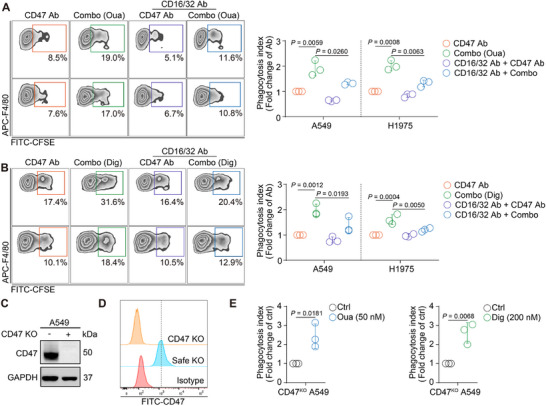
Fc‐FcγR interaction by the combination strategy partially enhanced the phagocytosis. A,B) Macrophages were pre‐incubated with anti‐CD16/32 antibody to block Fc‐FcγR interaction for 0.5 h, and cancer cells with or without ouabain C) or digoxin D) treatment were then added into the co‐culture systems. The phagocytosis was then tested by FACS after 3 h. Representative FACS results of the phagocytosis assay are presented in the left panel. (n = 3). (C) The protein expression level of CD47 was detected after CD47 was depleted in A549 cells. (D) The cell surface expression level of CD47 was detected in CD47 safety KO cell line and cd47 KO cell line. E) CD47 KO A549 cells was pretreated with 50 nM ouabain (left) or 200 nM digoxin (right) for 24 h, and these cells were then added into the co‐culture system. The phagocytosis rate was detected by FACS. (n = 3).

It is speculated that the detailed mechanism by which this combo strategy enhanced phagocytosis relied on the blockade of “don't eat me” signal transduction and the co‐stimulation of “eat me” signals. The ADCP effect, which is mediated by Fc‐Fc gamma receptor (FcγR) binding to an antibody, is a significant contributor to the increased phagocytosis observed following CD47 antibody treatment.^[^ ^39^ ^]^ Our findings indicated that CGs upregulated CD47 expression, which might result in an increased Fc‐FcγR interaction (Figure S3A,B, Supporting Information). Furthermore, blocking FcγR on macrophages with an anti‐CD16/32 antibody significantly reversed the enhanced phagocytosis mediated by the combo strategies with either ouabain or digoxin (**Figure** **3**A,B). Additionally, CG was also found to enhance the effect of cetuximab (EGFR antibody)‐mediated ADCP (Figure. S3C—E, Supporting Information). However, the magnitude of this phagocytic effect was significantly lower than that observed with the CG combined with CD47 antibody (Figure S3E, Supporting Information). These findings further corroborated the role of Fc‐FcγR mediated ADCP in the combo strategy and, more importantly, underscored the critical contribution of antibody targeting CD47 (but not other signals) within the combination approach.

Further analysis revealed that the anti‐CD16/32 antibody could not completely abrogate the increase in phagocytosis. To fully exclude the ADCP effects mediated by CD47 antibody, CD47 was knocked out (Figure 3C,D), and further examination of changes in phagocytosis was performed after ouabain or digoxin treatment. The results of the phagocytosis assay showed that treatment with ouabain and digoxin increased the phagocytosis rate in CD47 KO cells (Figure 3E). Therefore, other additional phagocytosis‐related signals might mediate the regulation of macrophage phagocytosis by this combination strategy.

### 2.4 Combination Treatment with CGs Elevated Ecto‐CRT to Promote Phagocytosis

**Figure 4:**
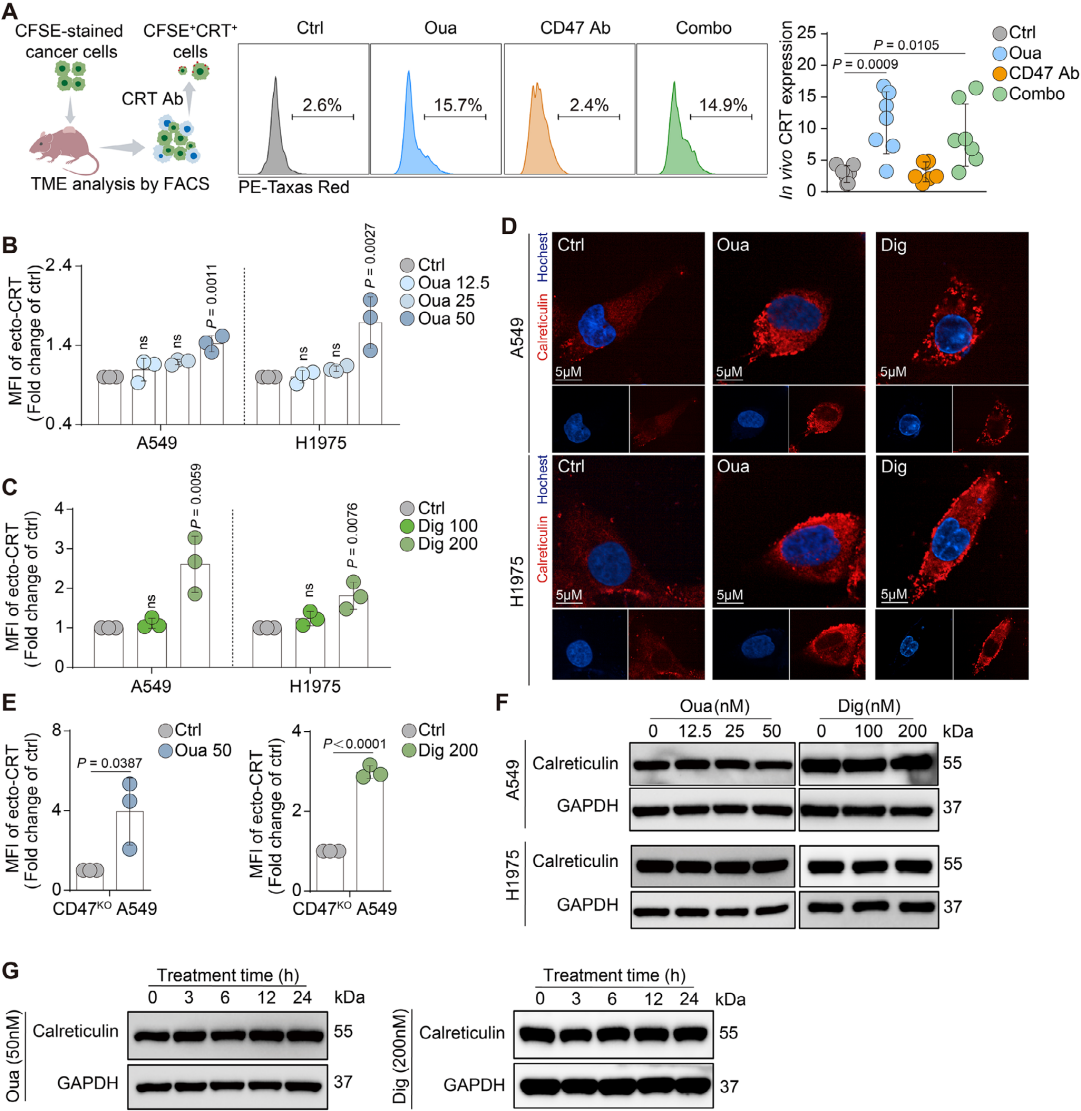
CGs increased ecto‐CRT level both in vitro and in vivo. A) The in vivo exploration of CRT in TME used the same tumor tissue samples in the in vivo phagocytosis assay. CFSE‐stained cancer cells were further conjugated with AF594‐labeled CRT staining. The CFSE^+^CRT^+^ cell population was detected by flow cytometry. (n = 7). B) The ecto‐CRT expression was detected after 12.5, 25, 50 nM ouabain treatment in A549 and H1975 cells for 24 h. (n = 3). C) The ecto‐CRT expression was detected after 100, 200 nM digoxin treatment in A549 and H1975 cells for 24 h. (n = 3). D) Cancer cells were incubated with 50 nM ouabain or 200 nM digoxin after 24 h, cells were fixed and CRT expression was then tested by IF. (scale bar: 5 µm) E) 50 nM Ouabain or 200 nM digoxin was used to treat CD47 KO A549 cells, and the ecto‐CRT was measured by FACS. (n = 3). F) Total protein level of CRT of A549 and H1975 cells treated with different concentration of ouabain or digoxin was analyzed by western blot. G) Total protein level of CRT of A549 cells with different time points of ouabain or digoxin treatment was detected. ns, no significance.

CGs have also been previously identified as inducers of immunogenic cell death (ICD),^[^ ^40^, ^41^ ^]^ in which the translocation of calreticulin (CRT) to the cell surface is one of the “hallmarks” of ICD.^[^ ^42^ ^]^ Moreover, surface‐expressed CRT (ecto‐CRT) is considered an “eat‐me” signal for macrophages during phagocytosis.^[^ ^43^ ^]^ Therefore, it was hypothesized that ouabain and digoxin might further enhance phagocytosis by upregulating ecto‐CRT expression. This hypothesis was validated in the in vivo experiments, where CRT‐positive cancer cells population in the TME were significantly increased in both the ouabain monotherapy group and the combo group (**Figure** **4**A). Moreover, treating lung cancer cells with different concentrations of ouabain and digoxin for 24 h resulted in increased ecto‐CRT expression with 50 nM ouabain and 200 nM digoxin (Figure 4B,C). The immunofluorescence results also indicated that ouabain and digoxin significantly enhanced ecto‐CRT expression (Figure 4D). Further surface detection of ecto‐CRT by FACS in CD47 KO cells also revealed that CG induced ecto‐CRT expression (Figure 4E). However, CGs treatment did not change the total CRT protein level (Figure 4F,G).

**Figure 5:**
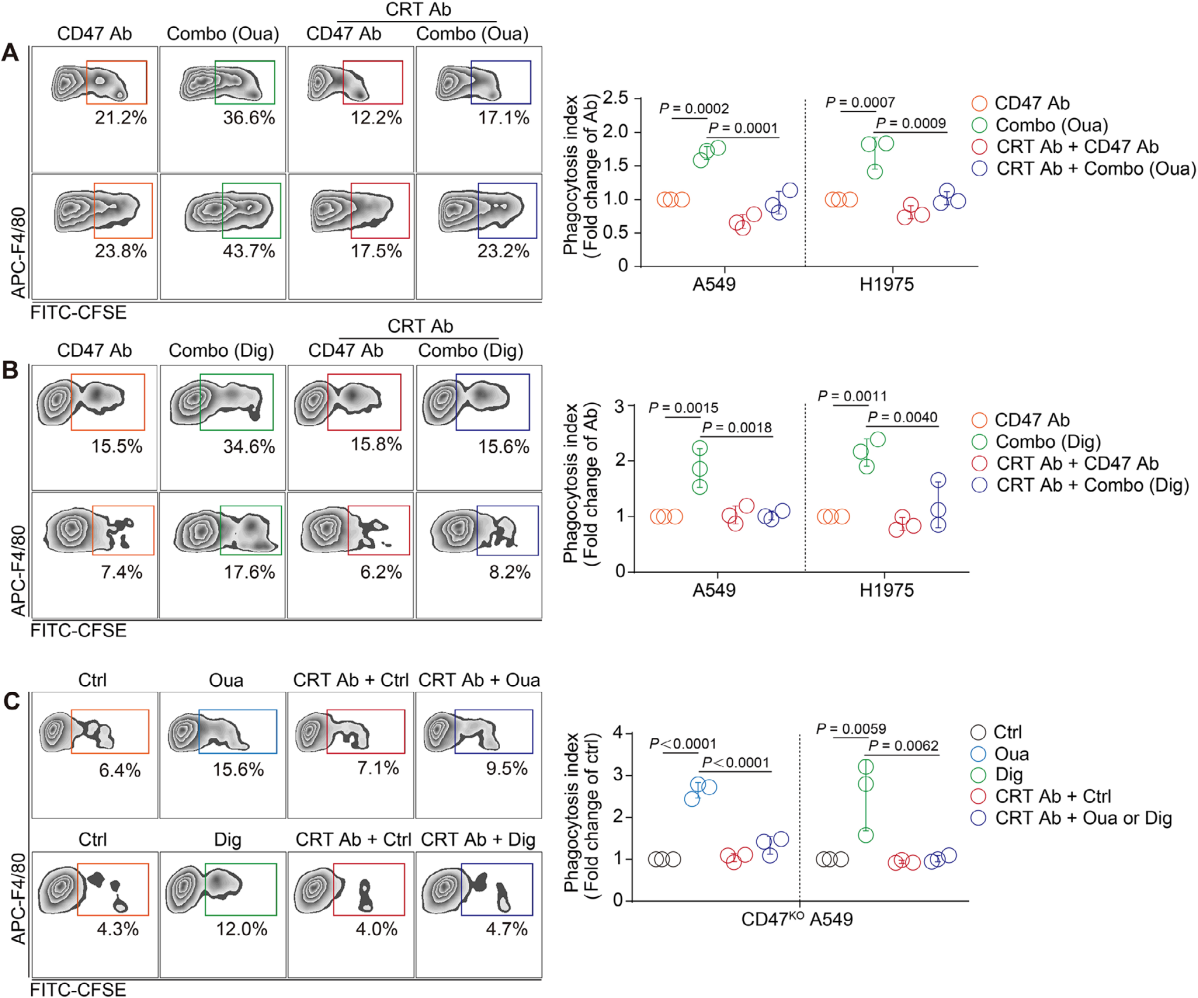
CRT antibody reversed the phagocytosis of combo treatment. A,B) A549 or H1975 cells pretreated with ouabain (A) or digoxin (B) were incubated with CRT Ab for 0.5 h. After the blockade, cancer cells were added to macrophages for 3 h, and the phagocytosis index was detected by FACS. Representative FACS results of the phagocytosis assay are presented in the left panel. (n = 3). C) CD47 KO A549 cells pretreated with ouabain (left) or digoxin (right) were incubated with CRT Ab for 0.5 h, after the blockade, cancer cells were added into macrophages for 3 h, and the phagocytosis index was tested by FACS. Representative FACS results of the phagocytosis assay are presented in the left panel. (n = 3).

To further confirm the important role of ecto‐CRT in enhanced phagocytosis, cancer cells pretreated with CGs for 24 h were co‐cultured with a CRT blocking antibody (CRT Ab) before being added to macrophages. The increase in phagocytosis caused by the combo strategy was significantly reversed (**Figure** **5**A,B). Similar reversal of phagocytosis was also observed in CD47 KO cells treated with ouabain and digoxin (Figure 5C). These results suggest that, in addition to the ADCP effect induced by the combo strategy, the increase in ecto‐CRT expression was a major contributor to the enhanced phagocytotic activity of macrophages.

### 2.5 CGs Induced Ecto‐CRT Expression via ER‐Ca^2^⁺ Disruption and PERK Activation

**Figure 6:**
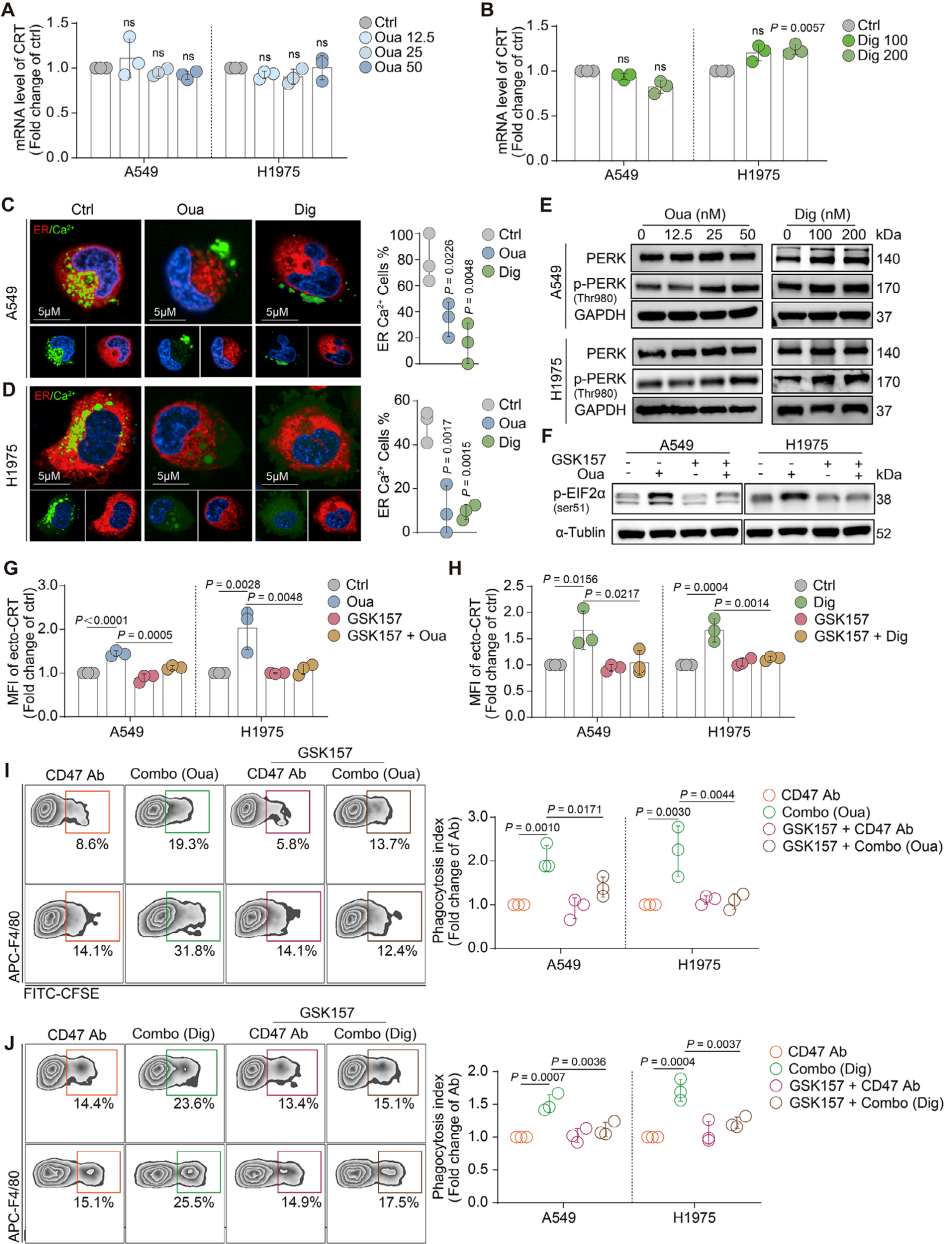
CGs disrupted ER‐Ca^2+^ hemostasis, activated PERK, and led CRT translocated to the cell surface. A,B) mRNA level of CRT was analyzed by qPCR in A549 and H1975 cells with different concentrations of ouabain or digoxin treatment. (n = 3). C,D) Representative confocal images of ER‐Ca^2+^ after short CGs stimulation was shown. Cancer cells were stained with ER‐tracker red (red) and Fluo‐4 AM (green) to track Ca^2+^ in the ER (ER‐Ca^2+^). Hoechst 33342 (shown in blue) was used to stain cell nuclear. After the staining, cancer cells were treated with ouabain or digoxin for 1 h, the ER‐Ca^2+^ was imaged by confocal. And the ER‐Ca^2+^ cells were counted in different views by 3‐time independent experiments. (scale bar: 5 µm). E) Cancer cells were treated with different concentrations of ouabain or digoxin. WB was used to detect pPERK, tPERK and GAPDH expression after CGs treatment. F) Western blot was used to detect the inhibiting effect of GSK2656157 (GSK157). p‐EIF2α was the downstream protein of PERK pathway. G,H) PERK inhibitor, GSK157, was used to inhibit PERK activation before the cancer cells were treated with ouabain (left) or digoxin (right). The ecto‐CRT was then tested by FACS. (n = 3). (I, J) A549 or H1975 cells was pretreated with GSK157 for 1 h, then ouabain I) or digoxin J) were added into the system for 24 h. After compounds treatment, cancer cells were incubated with macrophages for 3 h, and the phagocytosis index was tested by FACS and analyzed by FlowJo. Representative FACS results of the phagocytosis assay are presented in the left panel. (n = 3). ns, no significance.

Notably, cancer cells treated with ouabain or digoxin were analyzed by qPCR, and the results showed that neither CG significantly altered CRT mRNA levels (**Figure** **6**A,B). Thus, the mechanism by which CGs facilitate the translocation of CRT to the membrane without modulating CRT expression was investigated. In our experiments, both ouabain and digoxin were effective at nanomolar concentrations. Studies have shown that nanomolar levels of CG bind to ATP1A1 (the α1 subunit of Na^+^K^+^ATPase), leading to the secretion of IP3, which binds to IP3R on the endoplasmic reticulum (ER) membrane, opening ER‐Ca^2+^ release channels and leading to Ca^2+^ oscillation.^[^ ^44^, ^45^ ^]^ This action results in a significant influx of ER Ca^2+^ into the cytoplasm, causing Ca^2+^ homeostasis imbalance. To investigate whether similar phenomena also occur in CG‐treated cancer cells, ER‐tracker red dye and a Ca^2+^ indicator were used to track ER‐Ca^2+^ changes in live cancer cells with or without CG treatment. The obtained images suggested that CG treatment led to ER‐Ca^2+^ release into the cytoplasm, although not all cells in the field of view presented similar phenomena (Figure 6C,D).

In general, the disruption of Ca^2+^ homeostasis in the ER is associated with ER stress.^[^ ^46^ ^]^ Ouabain and digoxin activated PERK significantly (Figure 6E). The PERK inhibitor, GSK2656157 (GSK157), was used to pretreat the cells, which were then subjected to two CG treatments. GSK157 pretreatment combined with CG treatment did not lead to the phosphorylation of eIF2α, which is a downstream protein of the PERK pathway (Figure 6F). In A549 and H1975 cells, PERK inhibition reversed the upregulation of ecto‐CRT induced by CGs (Figure 6G,H; Figure S4A, Supporting Information). This finding prompted us to further investigate whether this inhibition could reverse macrophage phagocytosis. Pretreating A549 and H1975 cells with GSK157 followed by ouabain or digoxin treatment, significantly reversed the phagocytosis effect in the coculture system (Figure 6I,J). These results suggest that the primary step by which CGs lead to CRT translocation to the membrane may involve disrupting ER‐Ca^2+^ homeostasis, potentially inducing the activation of the PERK pathway. Inhibition of PERK could abrogate activation of the downstream pathway and mitigate CRT movement from the ER to the cell membrane.

### 2.6 Inhibition of ER‐Golgi Translocation Reversed the Increase in Phagocytosis Induced by the Combo Strategy

**Figure 7:**
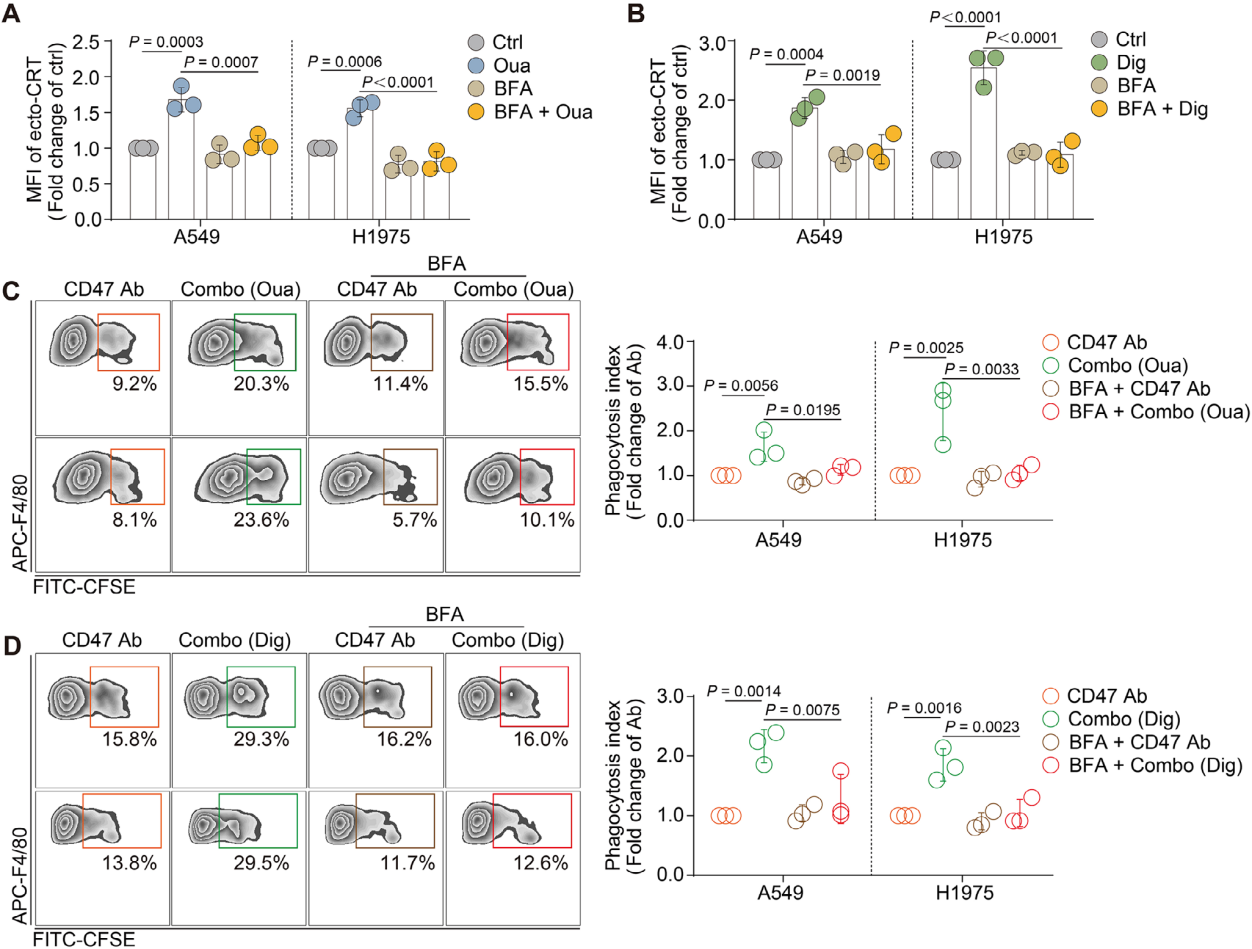
CGs‐induced CRT transported from the ER through the Golgi apparatus. A) ER‐Golgi protein trafficking inhibitor, BFA, was added to cancer cells for 1 h, followed by treatment with ouabain (left) or digoxin (right). The ecto‐CRT was then tested by FACS assay and showed as a fold change of ctrl group. (n = 3). C,D) A549 or H1975 cells were pretreated with BFA for 1 h, followed by ouabain (C) or digoxin (D) treatment for 24 h. Cancer cells after compounds treatment were incubated with macrophages for 3 h, the phagocytosis index was tested by FACS and analyzed by FlowJo. Representative FACS results of the phagocytosis assay are presented in the left panel. (n = 3).

Next, we explored whether the increase in ecto‐CRT induced by CGs also needed ER‐to‐Golgi apparatus translocation.^[^ ^47^ ^]^ Brefeldin A (BFA) is a fungal macrocyclic lactone and a potent, reversible inhibitor of intracellular vesicle formation and protein trafficking between the ER and the Golgi apparatus.^[^ ^48^ ^]^ After cancer cells were pretreated with BFA for 3 h, the cells were then treated with ouabain or digoxin for 24 h, followed by flow cytometry analysis and IF detection. The results revealed that ecto‐CRT no longer increased (**Figure** **7**A,B; Figure S4B, Supporting Information). To determine whether the reversal of ecto‐CRT also implies a reversal of phagocytosis by macrophages, corresponding phagocytosis assays were conducted. After pretreatment with BFA, subsequent treatment with CGs combined with CD47 antibody did not further enhance phagocytosis in both A549 and H1975‐based coculture system (Figure 7C,D). These results suggested that the ecto‐CRT induced by CGs reached the membrane via ER‐Golgi apparatus translocation. Inhibiting this pathway reversed the phagocytosis of the combo strategy.

### 2.7 Exo70 Mediated the Exocytosis of CRT from Golgi to Cell Surface

**Figure 8:**
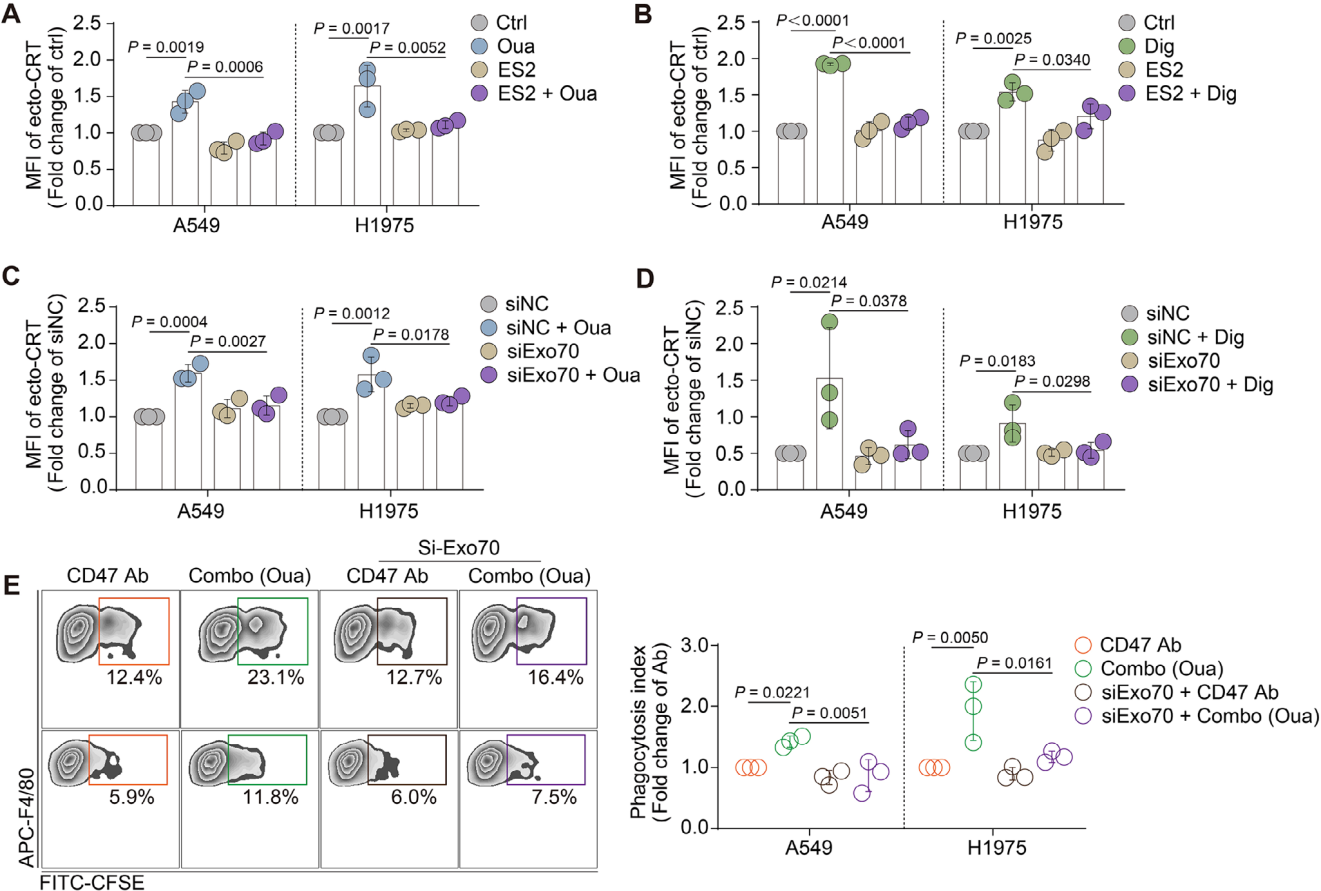
Exo70‐mediated exocytosis participated in CRT translocation to cell surface. A,B) A549 or H1975 cells were pretreated with the exocytosis inhibitor, ES2, followed by 24 h ouabain (A) and digoxin (B) treatment. The ecto‐CRT was then tested by FACS assay and showed as fold change of ctrl group. (n = 3). C,D) Cancer cells were transfected with si‐exo70 for 48 h, and ouabain was added into cells for another 24 h. The ecto‐CRT was then tested by FACS assay and showed as fold change of si‐NC group. (n = 3). E) Cancer cells were transfected with si‐exo70 for 48 h, followed by ouabain treatment for another 24 h. Then, cells were added to macrophages for 3 h, the phagocytosis index was tested by FACS and analyzed by FlowJo. Representative FACS results of the phagocytosis assay are presented in the left panel. (n = 3).

Secretory vesicles form from the trans‐Golgi network, and we thus explored the next step for CRT to reach the cell surface is through exocytosis.^[^ ^49^ ^]^ To this end, A549 and H1975 cells were pretreated with endosidin‐2 (ES2), an exocytosis inhibitor. The FACS results indicated that ES2 significantly reversed the CG‐induced upregulation of ecto‐CRT (**Figure** **8**A, B). Previous studies have reported that ES2 targets *exo70*.^50^ Therefore, siRNA was used to target *exo70*, and the knockdown efficiency of the SiRNA is shown in Figure S4C (Supporting Information). After knockdown of *exo70*, the effect was similar to that of ES2, with ecto‐CRT being reversed (Figure 8C,D). Additionally, the phagocytosis assay revealed that *exo70* knockdown partially reversed the increased phagocytosis induced by the strategy (Figure 8E). This phenomenon demonstrated that Exo70 is an essential protein required for exocytosis‐mediated CRT translocation from the Golgi apparatus to the cell surface.

## 3 Discussion

Our study provides the first evidence that the co‐administration of CD47 antibody and CGs has a significant anti‐cancer effect on lung cancer models. To identify novel combination strategies targeting CD47, a macrophage killing‐based evaluation system was developed, encompassing short‐term phagocytosis assays, long‐term assessments of residual tumor cells, and in vivo phagocytosis analyses (Figure 1). Although some screened compounds exhibited high phagocytosis rates at 3 h, they resulted in substantial residual tumor cell populations. This observation indicated that relying on the phagocytosis index as a single parameter for anti‐cancer screening in our system may not yield optimal outcomes. While positive results were obtained through the screening, the number of screening compounds could be further expanded to identify additional combinations that effectively enhance macrophage‐mediated anti‐tumor activity. Furthermore, it is believed that the emerging phagocytosis checkpoints will make our screening system applicable to the exploration of all strategies targeting macrophage phagocytosis, extending beyond CD47‐targeted therapies.^[^ ^15^ ^]^

Many preclinical studies have recently focused on discovering the multifaceted anti‐cancer effects of CGs as monotherapies.^[^ ^51^ ^]^ Additionally, at least 7 ongoing clinical trials have evaluated the individual effects of CGs on cancer patients.^[^ ^29^ ^]^ Repurposing CGs from heart failure treatment to cancer therapy is a highly promising direction.^[^ ^29^ ^]^ Trying different strategies will further prompt its development. Notably, combo strategies involving CG and immunotherapy have rarely been explored in clinical trials or preclinical studies. Our strategy demonstrates the feasibility of CGs and immunotherapy. It has been shown to induce complete tumor regression in a lung cancer model, indicating its great translational potential. Furthermore, both ouabain and digoxin enhanced the anti‐cancer effect of CD47 antibody in this study. Other CGs, such as digitoxin, oleandrin, bufalin, and cinobufagin, are also likely to boost the efficacy of CD47 antibody since their mechanisms of action are similar to those of ouabain and digoxin.^[^ ^40^, ^41^ ^]^

The narrow therapeutic window of CGs has long been a concern, although our in vivo data confirm their safety to a certain extent.^[^ ^52^ ^]^ From the perspective of optimizing clinical translation, it is crucial to identify the effective concentrations of CGs in patient with cancer and distinguish them from their toxic concentrations. Several clinical trials have commenced to assess the safety and tolerance of different doses of CGs in cancer patients (NCT02212639, NCT00017446, NCT02530398, NCT04621669, and NCT05828303), including the pharmacokinetic data of CGs in these patients concurrently receiving other treatments.^[^ ^29^ ^]^ More importantly, in our strategy, both the apoptosis evaluation assay and phagocytosis assay indicated that the involvement of CD47 antibody enables CGs to regulate the phagocytosis of cancer cells rather than directly killing them at the nanomolar level. Therefore, the concentrations of CGs could be further optimized in the future to achieve the clinical translation of this strategy. Additionally, considering the safety profile of CGs, finding regimens to assist CGs in precisely targeting tumor sites is another approach.^[^ ^53^, ^54^, ^55^ ^]^ Chen et al. prepared folate and transferrin (due to the high expression of their receptors in cancer cells) to co‐modify liposome delivery systems containing CGs. The results showed that the uptake of targeted liposomes by cancer cells increased 6‐fold, indicating stronger anti‐cancer effects.^[^ ^56^ ^]^ In addition, studies have developed a biomimetic nanosystem of cancer cell membranes coated with digoxin or a combination of ursodeoxycholic acid and p‐biguanylbenzoic acid (both of which have specific targeting effects on hepatocellular carcinoma) that combines albumin submicrospheres with bufalin to achieve cancer cell‐targeted delivery.^[^ ^57^ ^]^ On the basis of our findings, future strategies may involve the combination of CD47 antibody and other immune checkpoint antibodies), or antibody‐drug conjugates (ADCs) while leveraging the high expression of CD47 and/or tumor‐specific proteins to increase the precision of tumor targeting.^[^ ^58^ ^]^ This approach could facilitate the development of more efficient delivery systems aimed at cancer cells, simultaneously enabling CGs to achieve their potent efficacy against cancer.^[^ ^59^, ^60^ ^]^

Mechanistically, treatment with EGFR antibody alone (which primarily activated the “eat me” signal, Fc‐FcγR interactions) resulted in only a modest increase in phagocytosis (Figure S3B, Supporting Information).^[^ ^61^ ^]^ Similarly, a slight enhancement of phagocytic activity was observed in CD47 KO cells (which solely block the “don't eat me” signal). More pronounced effects were achieved with combination strategies, such as an anti‐EGFR antibody combined with a CG, which simultaneously engages Fc‐FcγR interactions and induce ecto‐CRT exposure (another “eat me” signal), or with CD47 KO cells treated with CGs, which target both CD47 blockade and ecto‐CRT. In contrast, the more effective phagocytosis enhancement occurred using CD47 antibody combined with CGs (Figure S3C, Supporting Information). These findings validate CD47 is still one of the most crucial brakes on phagocytosis in lung cancer, and that a therapeutic strategy combining blockade of the “don't eat me” signal (CD47) with boosting both “eat me” signals (Fc‐FcγR and ecto‐CRT) is a feasible way to maximize the macrophage clearance in cancer cells.^[^ ^62^ ^]^

Importantly, our results have confirmed the importance of CRT as an “eat‐me” signal. CGs can upregulate CRT expression both in vitro and in vivo, and when the CRT antibody is applied, the phagocytic activity stimulated by CGs is nearly completely inhibited. While CRT has been extensively characterized as a pro‐phagocytic signal,^[^ ^63^, ^64^ ^]^ the identity of its receptor on macrophages remains unclear in this study. Some studies suggest that LRP‐1 may serve as a receptor for CRT on macrophages.^[^ ^43^, ^65^ ^]^ However, its role in promoting phagocytosis remains to be further validated.^[^ ^66^, ^67^ ^]^ Moreover, CRT has been identified to interact with additional ligands, such as C1q and phosphatidylserine (PS).^[^ ^68^, ^69^, ^70^ ^]^ Therefore, confirming the specific CRT ligands involved in our combo strategy represents an important avenue for future investigations.^[^ ^71^ ^]^

Although ouabain has been reported to modulate CD40 / CD80 in human monocytes,^[^ ^72^ ^]^ our in vitro and in vivo studies did not reveal ouabain‐induced repolarization of mouse‐derived macrophages (Figure S2, Supporting Information). This discrepancy may stem from differences in macrophage origin. Consequently, in our experimental system, the effect of CGs combined with CD47 antibody depended primarily on macrophage‐mediated phagocytosis, not macrophage repolarization.^[^ ^73^ ^]^ Moreover, given the complexity of the TME, additional immune cells may also contribute. Considering the increased expression of ecto‐CRT on cancer cells that we identified, NK cells may also participate through the NKp46‐ecto‐CRT axis.^[^ ^74^, ^75^ ^]^ Although our T‐cell proliferation assays showed that enhanced phagocytosis by combo treatment did not expand T cells population (data not show), macrophages can serve as antigen‐presenting cells (APCs) post‐phagocytosis,^[^ ^76^ ^]^ potentially promoting other antigen‐specific T‐cell responses.^[^ ^77^ ^]^ Therefore, our study still lays the foundation for the subsequent involvement of adaptive immune response in immune‐competent mouse model.

**Figure 9:**
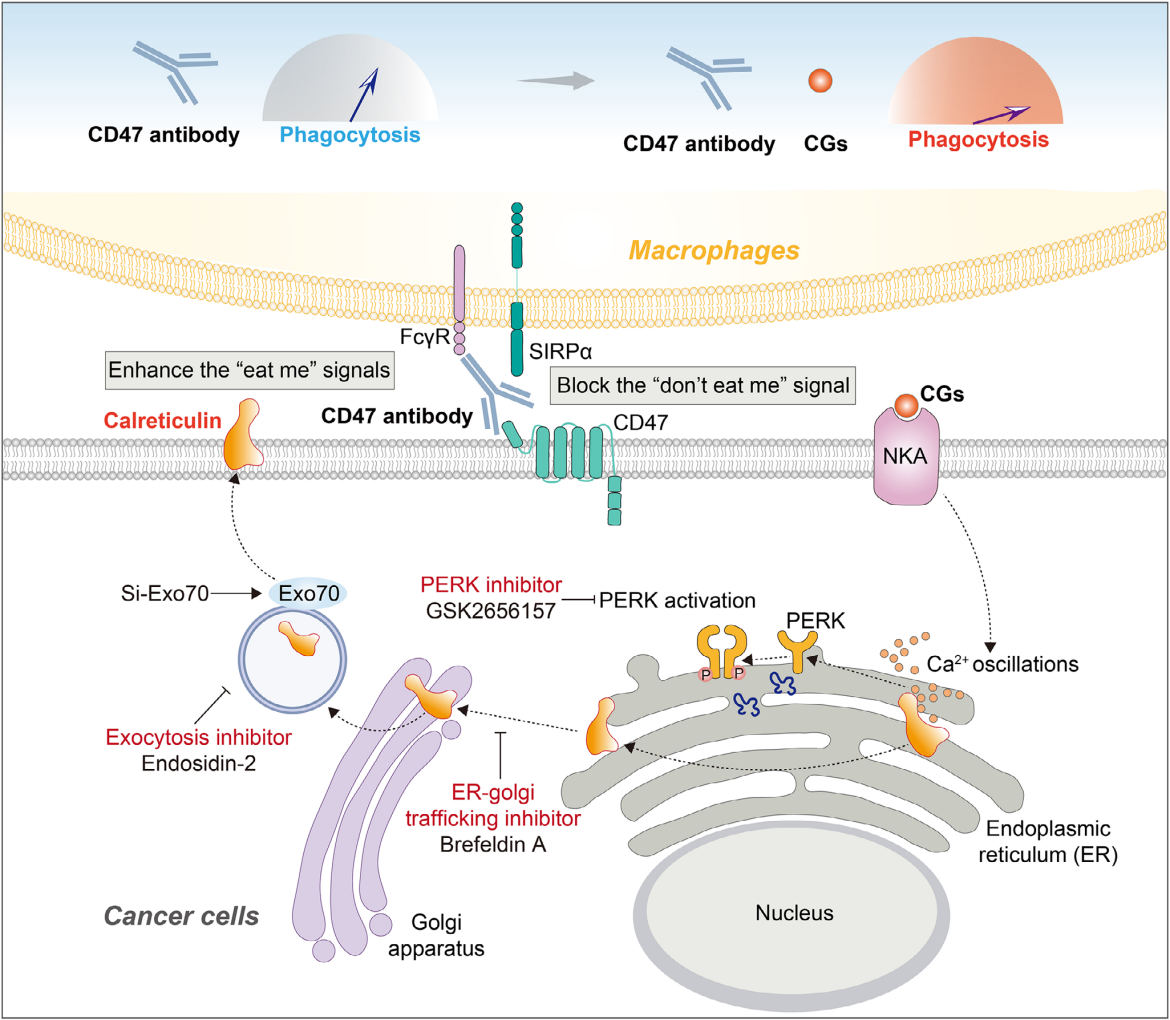
Schematic drawing showing the detailed mechanism. CGs combined with CD47 antibody enhanced phagocytosis by blocking the “don't eat me” signal (CD47) and increasing the “eat me” signals (CRT and Fc‐FcγR interaction). The CGs‐induced CRT exposure on the cell surface was through disruption of ER‐Ca^2+^ hemostasis. The PERK inhibitor GSK2656157 effectively reversed CGs‐induced CRT upregulation and phagocytosis enhancement. Furthermore, the ER‐Golgi protein trafficking inhibitor BFA and siRNA targeting the exocytosis protein Exo70 also mitigated CGs‐induced CRT expression and phagocytosis. Therefore, it was speculated that after CGs treatment, CRT release from ER to Golgi, along with PERK activation. Subsequently, CRT was then translocated to the cell surface via Exo70‐mediated exocytosis.

CGs were found to have no effect on the mRNA level or total protein level of CRT (Figure 4, 6). These findings suggest that the mechanism through which CGs induced ecto‐CRT may partially resemble that of classical ICD inducers, such as oxaliplatin.^[^ ^78^ ^]^ Both PERK activation and ER‐Golgi translocation, which mediate CG‐induced upregulation of ecto‐CRT, were observed in the present study. In contrast, the functional assay performed in this study further demonstrated that a PERK inhibitor (GSK157) and an ER‒Golgi transport inhibitor (BFA) further reversed phagocytosis caused by combo treatments, underscoring the pivotal role of ecto‐CRT in this process.^[^ ^50^ ^]^ Additionally, a previously unrecognized function of Exo70 in CRT translocation was unveiled. Exo70 is the target of the exocytosis inhibitor ES2 and also a key component of the exocyst complex involved in exocytosis.^[^ ^50^ ^]^ This protein might facilitate the process of CRT translocation from the Golgi apparatus to the membrane induced by CGs (**Figure** **9**). However, whether other ecto‐CRT inducers exhibit similar phenomena remains unknown.

In summary, our study revealed that CGs significantly enhanced the anti‐lung cancer effect of CD47 antibody. The combo strategy significantly increased the phagocytic effect of macrophages both in vivo and in vitro. More specifically, the increase in phagocytic effect was due to the enhancement of “eat me” signals, Fc‐FcγR and CRT, induced by CGs combined with CD47 antibody. The entire process by which CGs induce CRT translocation from ER to cell surface has been revealed. This study offers novel insights into the potential repurposing of CGs for anti‐cancer applications within the framework of macrophage‐related immune response, and establishes a conceptual basis for the future clinical translation of CD47 antibody therapies.

## 4 Experimental Section

### Reagents

Ouabain octahydrate (# HY‐B0542), digoxin (# HY‐B1049), GSK2656157 (# HY‐13820), brefeldin A (# HY‐16592), and endosdin‐2 (# HY‐120821) were purchased from MedChemExpress (Shanghai, China). RPMI 1640 medium, DMEM, foetal bovine serum (FBS), penicillin‐streptomycin (100×), phosphate‐buffered saline (PBS), trypsin, collagenase IV, and DNase I (used for single cell suspension preparation) were purchased from Gibco (Carlsbad, CA, USA). The LIVE/DEAD Fixable Yellow Dead Cell Stain Kit, LIVE/DEAD™ Fixable Near‐IR Dead Cell Stain Kit, FoxP3/Transcription Factor Staining Buffer Set, and Intracellular (IC) Fixation Buffer were acquired from Invitrogen (Carlsbad, CA, USA).

### Cell Culture

The human NSCLC cells lines, A549 and NCI‐H1975 (American Type Culture Collection, ATCC, Rockville, MD, USA) were cultured in RPMI‐1640 medium (Gibco, Carlsbad, CA, USA) with 10% (v/v) FBS and 1% (v/v) penicillin (100 units/ml)–streptomycin (100 µg ml^−1^). CD47 knockout cells (A549/CD47KO) were established through CRISPR Cas9‐mediated genome editing, and the process of generating a knockout cell line was described previously.^[^ ^26^ ^]^ Briefly, A549 cells were transfected with the CD47 KO Lenti‐CRISPR v2 plasmid (obtained from Addgene, Watertown, MA, USA). The transfection was carried out using TurboFect™ Transfection Reagent from Invitrogen following the manufacturer's instructions. Single‐cell clones were selected by means of the dilution method and then expanded to obtain CD47 knockout cell populations. All the cell lines were cultured with 5% CO_2_ at 37 °C.

### Mice

Three‐week‐old BALB/c mice and five‐week‐old BALB/c nude mice were obtained and maintained at the specific‐pathogen‐free animal facility at the University of Macau. All animal experiments were conducted in accordance with the ethical guidelines set by the Animal Ethics Committee of the University of Macau and complied with the principles regarding the Care and Use of Laboratory Animals (approved ethics ID: UMARE‐008‐2023 and UMARE‐041‐2024).

### Mouse Xenograft Model

First, A549 (1 × 10^7^ cells) and NCI‐H1975 cells (2 × 10^6^ cells) were injected subcutaneously into the right back of each BALB/c nude mouse. After the average tumor volume reached 80 mm^3^, the mice with similar body weights were randomly divided into the following treatment groups: (1) Solvent contorl group, (2) Oua treatment group (1 mg kg^−1^, *i.t*. injection, three times a week), (3) CD47 antibody treatment group (200 µg per mouse, *i.p*. injection, 24 h after oua treatment), and (4) Combo group of Oua and CD47 Ab. Oua and CD47 antibody B6H12 (BioXcell, West Lebanon, NH, USA, # BE0019‐1) were diluted with phosphate‐buffered saline to achieve the final concentration, and 0.1 mL was injected into each mouse. Tumor sizes were detected every 2 days and calculated with the formula (width × length)/2. Tumor tissues and mouse serum were harvested after the indicated treatment period. Tissues for the H&E staining were obtained from Scientist Biotechnology (Chengdu, China). Serum alanine aminotransferase (ALT) / aspartate aminotransferase (AST), creatine kinase (CK), and lactate dehydrogenase (LDH) levels were measured by Bestest Biotechnology Co., Ltd (Zhuhai, China). Tissues for TME analysis were digested with collagenase IV (1 mg mL^−1^) and deoxyribonuclease I (0.1 mg mL^−1^). Following centrifugation, the fragments were filtered to generate a single‐cell suspension, which was then subjected to LIVE/DEAD fixable dye staining overnight. After the Fc receptors were blocked, the suspended cells were incubated with specifically diluted antibodies for an additional 30 min and analyzed by flow cytometry (BD LSRFortessa, Franklin Lakes, NJ, USA).

### Phagocytosis Assay

An in vitro phagocytosis assay was performed with a co‐culture system of bone marrow‐derived macrophages (BMDMs) from mice and human cancer cells. BMDMs from the femora and tibiae of BALB/c mice were generated by culturing them in 10% FBS DMEM supplemented with 25% L929 supernatant for 7 days.^[^ ^79^ ^]^ BMDMs were plated in 24‐well plates at a density of 1.5 × 10^5^ cells per well and incubated overnight. To block Fc‐FcγR, BMDMs were pretreated with purified anti‐mouse CD16/32 antibody from BioLegend (San Diego, CA, USA, # 101301) for 0.5 h at 37 °C. For the blockade of ecto‐CRT, cancer cells treated with CGs were incubated with anti‐calreticulin antibody from Abcam (Cambridge, UK, # ab2907) for 1 h before their staining and addition to the co‐culture model. Cancer cells were stained with 2.5 µmol L^−1^ CFSE from Invitrogen (#C34554) at 37 °C for 10 min following the manufacturer's protocols. The working concentration of CD47 antibody, B6H12 in our co‐culture model was 10 µg ml^−1^. For anti‐EGFR antibody, cetuximab, the working concentration was 2 µg ml^−1^. The cells were added to the macrophages (with a ratio of cancer cells: macrophages = 2:1) for another 3 h at 37 °C. The macrophages were stained with APC‐labelled anti‐F4/80 (BioLegend, #123116) and detected by flow cytometry (BD LSRFortessa). CFSE^+^ and F4/80^+^ double‐positive cells were identified as positive phagocytic cells, and the phagocytosis rate was calculated as (CFSE^+^F4/80^+^ cells / F4/80^+^ cells) × 100% and reported as the fold change of control group.

An in vivo phagocytosis assay was performed in A549‐bearing balb/c nude mice model.^[^ ^37^ ^]^ CFSE‐labelled A549 cells were inoculated subcutaneously into the right flank of the prepared mice. Once the average tumor volume was 80 mm^3^, the mice were randomly divided into four groups as the xenograft model. After the treatment, the cell suspension was filtered, washed three times with DMEM, and subsequently resuspended. The cell density was adjusted to 1 × 10⁶/100 µL using FACS buffer. The following fluorescent antibodies were used to label and identify tumour‐associated macrophages: anti‐mouse CD45 from Biolegend (APC/Cyanine7, #103116; Brilliant Violet 605, #103115) and anti‐mouse F4/80 (#123116) were obtained from BioLegend. Next, 0.5 µL of APC‐labelled anti‐mouse F4/80 antibody was added per 100 µL of the cell suspension, and the mixture was incubated at 4 °C for 30 min in the dark. The cell suspension was centrifuged at 300 × g for 5 min and resuspended in cold FACS buffer. Finally, flow cytometric data were obtained, and the data were analyzed with FlowJo v10 software (FlowJo LLC, Ashland, OR, USA). The gating strategy is shown in Figure S1A (Supporting Information). Notably, we also integrated and analyzed the flow cytometry results and revealed that the CFSE signal remained detectable across all treatment groups, supporting the feasibility of this assay (Figure S2B, Supporting Information).

### Screening of Compounds

The screening compounds were from the FDA‐approved drug library (MedChemExpress, Shanghai, China, #HY‐L022M). The screening system was based on the phagocytosis and killing effects of BMDMs. Briefly, mature BMDMs were digested with trypsin, stained with 2.5 µmol L^−1^ CFSE, and plated in 96‐well plates (at a density of 1.5 × 10^4^ cells per well) overnight. A549 cells that had been treated with different combinations of compounds and CD47 antibody were stained with Cell‐Tracker Orange CMTMR from Invitrogen (#C2927) at 37 °C for 10 min following the manufacturer's protocols. The co‐culture system was analysed by high‐content imaging after 3 h for detection of the phagocytosis rate and 48 h for detection of the remaining cancer cells by only showing the orange CMTMR‐stained A549 cells according to the HCI operation method.

### Western Blot

The process of Western blotting was described previously.^[^ ^79^ ^]^ Briefly, cellular proteins were obtained using RIPA lysis buffer (Beyotime, Shanghai, China), separated by SDS‐PAGE, and transferred onto PVDF membranes (Bio‐Rad, Hercules, CA, USA). After being incubated with blocking buffer, PVDF membranes were stained with specific primary antibodies (all of antibodies were from Cell Signaling Technology, CST, Beverly, MA, USA) for CD47 (#63000), calreticulin (#12238), PERK (#3192), p‐PERK (Thr980) (16F8) (#3179), p‐EIF2α (Ser51) (#3398), α‐Tubulin (#3873) and GAPDH (#2118). The membranes were washed with TBST and incubated with anti‐rabbit IgG and anti‐mouse HRP‐conjugated secondary antibodies. The membranes were exposed to an ECL Western blotting detection kit (GE Healthcare, Buckinghamshire, UK) and visualized with a ChemiDoc imaging system (Bio‐Rad, Hercules, CA, USA).

### Quantitative Real‐Time PCR

RNA from cancer cells was extracted using either TRIzol reagent from Life Technologies (Shanghai, China) or an RNAeasy™ Animal RNA Isolation Kit from Beyotime. cDNA synthesis was subsequently carried out using the RevertAid First Strand cDNA Synthesis Kit (Thermo Fisher Scientific, Waltham, MA, USA). Quantitative real‐time PCR (qPCR) was conducted on a QuantStudio™ 7 Flex Real‐Time PCR System (Thermo Fisher Scientific, Waltham, MA, USA). The sequences of the primers used for PCR were as follows: 5′‐CGCTTTTATGCTCTGTCGGC‐3′ (forward) and 5′‐CCACAGATGTCGGGACCAA‐3′ (reverse); exo70: 5′‐TGGCCGCAACCAAGATTTCATG‐3′ (forward) and 5′‐ GAGAAGTCGTGTCGCACAATGGC‐3′ (reverse); and β‐actin: 5′‐ GCGACACCCACTCCTCCACCTTT‐3′ (forward) and 5′‐ TGCTGTAGCCAAATTCGTTGTCATA‐3′ (reverse). The relative expression of calreticulin and exo70 was normalized to that of β‐actin, and 2^−ΔΔCT^ was used to calculate the relative gene expression.

### Small Interfering RNA (siRNA) Transfection

The negative control or siRNA targeting Exo70 was transfected into cells by using Lipofectamine 3000 Transfection Reagent from Invitrogen, according to the manufacturer's protocol. The sequences used were (5’‐UUCUCCGAACGUGUCACGUTT‐3’) for siNC, and (5’‐ GGTTAAAGGTGACTGATTA‐3’) for siExo70.

### Flow Cytometry Assay

For the detection of CRT in vitro and in vivo, cancer cells were incubated with the primary antibody, an anti‐calreticulin antibody (#ab2907), at room temperature in the dark for 45 min and then conjugated with an Alexa Fluor 594‐labelled goat anti‐rabbit IgG (H + L) secondary antibody from Biolegend (#A‐11008). The cells were subsequently washed with PBS three times and analysed by flow cytometry (BD LSRFortessa).

For the detection of CD47 and EGFR in vitro, cancer cells were incubated with a FITC‐conjugated anti‐human CD47 antibody (#CC2C6, Biolegend) or PE anti‐human EGFR antibody (#AY13, Biolegend) at room temperature in the dark for 1 h. For the detection of apoptosis, cancer cells with or without treatment were incubated with an Annexin V‐FITC Kit (C1062M, Beyotime) and propidium iodide (PI, 5 µg ml^−1^) for 15 min at room temperature in the dark and analysed by flow cytometry (BD LSRFortessa).

### Immunofluorescence

The immunofluorescence experiment was carried out in accordance with the manufacturer's instructions. Briefly, for the detection of ER‐calcium (ER‐Ca^2+^), the cells were treated with ouabain or digoxin for 1 h, and ER‐tracker red (Beyotime, #C1041S) was used to stain cells for 0.5 h followed by staining with fluo‐4 AM (Beyotime, # S1060) for another 0.5 h. The cells were then stained with Hoechst 33342 for 10 min and photographed using a Leica TCS SP8 microscope (manufactured in Solms, Germany).

For the detection of ecto‐CRT in cells, after being treated with CGs, cells were fixed using 4% paraformaldehyde for 30 min. Subsequently, the cells were blocked with 0.5% bovine serum albumin (BSA) for 1 h and then incubated with the antibody against calreticulin overnight at 4 °C. After that, the cells were stained with the anti‐rabbit IgG (Alexa Fluor 594 Conjugate, CST, #8889) for an additional 1 h. Following three washes with phosphate‐buffered saline (PBS), the cells were further stained with Hoechst 33342 for 10 min and photographed using a Leica TCS SP8 microscope.

### Statistical Analysis

Statistical analyses were performed using GraphPad Prism 7 (GraphPad Software Inc., San Diego, CA, USA). The statistical data are presented as the means ± standard error of the means (SEMs). Two‐tailed Student's t‐tests were used for comparisons between two groups. Comparisons involving more than two groups were analyzed using one‐way ANOVA. Two‐way ANOVA with a multiple‐comparisons test was used to analyze tumor growth curves, followed by Fisher's LSD test. *P* values < 0.05 were considered as statistically significant, and all *P* values are specified in the figure.

## Conflict of Interest

The authors declare no conflict of interest.

## Supporting information



Supporting Information

## Data Availability

The data that support the findings of this study are available in the supplementary material of this article.
