# Supplementary material for: Repurposing Cardiac Glycosides to Potentiate CD47 Blockade through Calreticulin‐mediated Phagocytic Effects for Lung Cancer Treatment
Source: Adv Sci (Weinh). 2025 Sep 23;12(46):e08245. doi: 10.1002/advs.202508245 (PMC12697853; doi:10.1002/advs.202508245)
Supplement: Supplementary file 1 — Supporting Information [file ADVS-12-e08245-s001.docx]

**
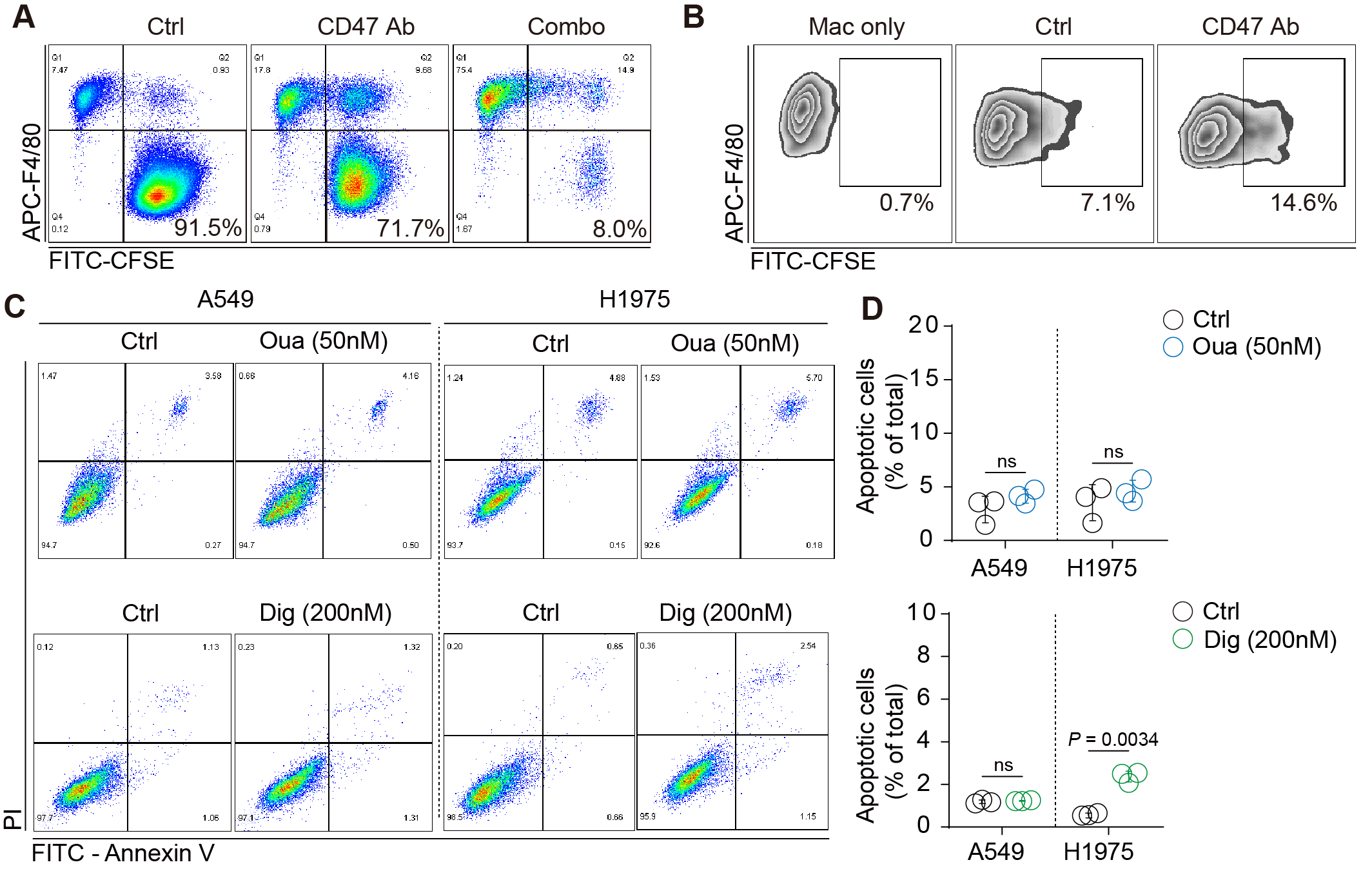
**

**Figure S1. The gating strategy of the function assay and** **the cytotoxic effect of CGs. (A)** CD47 antibody and combo (CD47 antibody + Oua) group were used to represent the percentage of remaining cells by FACS results after 48 h. **(B)** Mac only (macrophage only) group was used to set the phagocytosed cancer cells rate in FACS results after 3 h co-culture. **(C)** The representative flow cytometry data of the cytotoxic effect of ouabain or digoxin (left). The corresponding quantification of flow cytometry results of three-independent experiments (right). (n = 3).


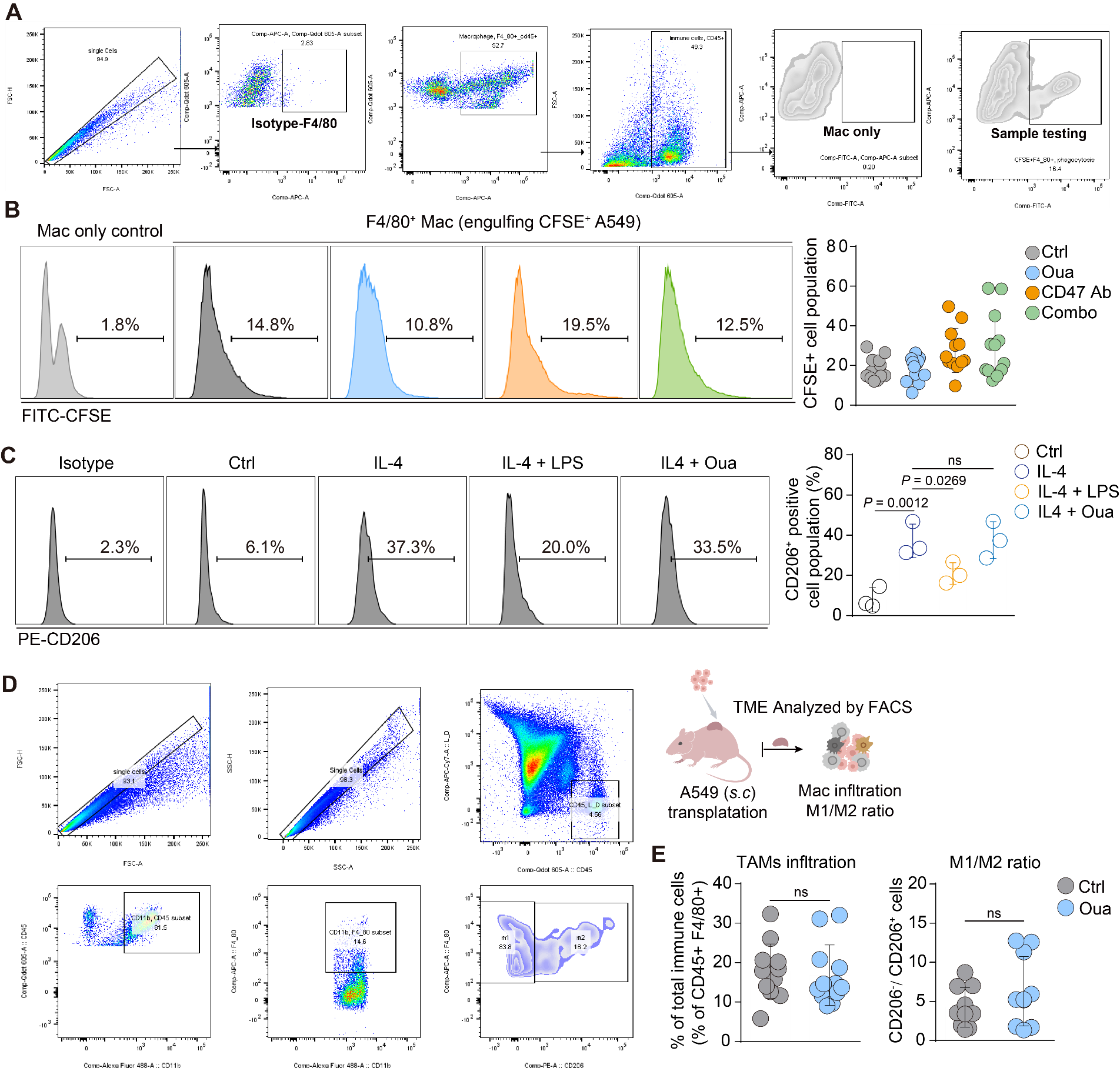


**Figure S2. The combo strategy exhibited modest immunomodulatory effects on macrophages. (A)** Gating strategy of detection of *in vivo* phagocytosis of TAMs in TME. **(B)** CFSE retention analysis in the *in vivo* phagocytosis assay. **(C)** The *in vitro* macrophage polarization assay was detected by FACS. (n = 3). **(D)** Gating strategy, study design and the result of macrophage infiltration and polarization *in vivo*. **(E)** The infiltration of CD45^+^CD11b^+^F4/80^+^ cells (left, n = 12) and the percentages of M2 type of macrophage (CD206^+^) cells in total macrophages (F4/80^+^) cells (right, n = 9) with or without ouabain treatment.


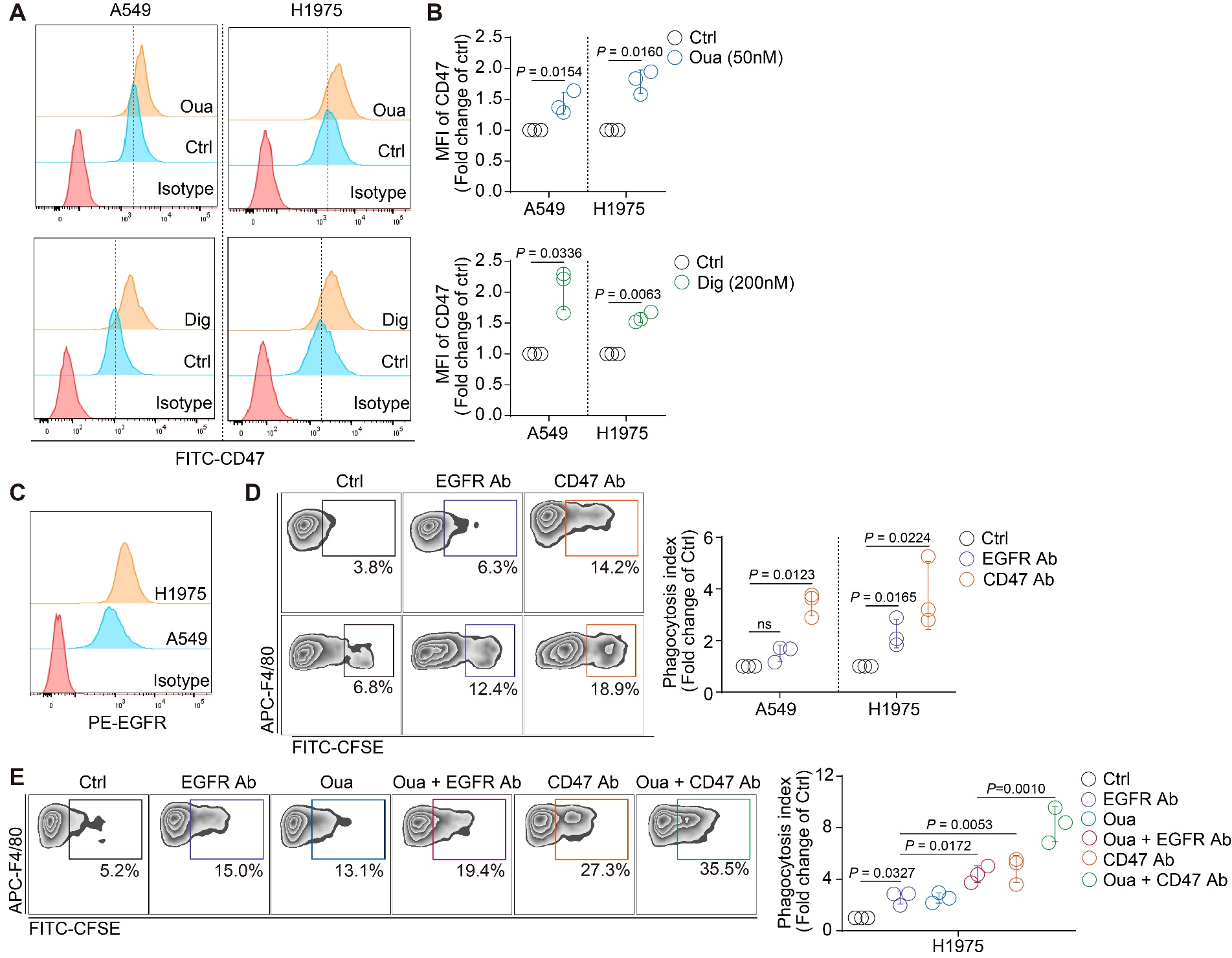


**Fig S3. CGs increased CD47 expression and the detection of ADCP-induced by EGFR antibody.** (A, B) The representative flow cytometry data of CD47 expression after 50 nM ouabain or 200 nM digoxin treatment (A). The corresponding quantification of flow cytometry results (B) (n = 3). (C) The EGFR expression of A549 and H1975 cells was detected by flow cytometry. (D) Cancer cells were added to macrophages for 3 h with or without EGFR antibody or CD47 antibody, the phagocytosis index was tested by FACS and analyzed by flowjo. Representative FACS results of the phagocytosis assay are presented in the left panel. (n = 3). (E) Macrophage were pretreated with 50 nM ouabain for 24 h and then incubated with H1975 cancer cells in the presence of EGFR antibody (or CD47 antibody) for 3 h. The phagocytosis index was detected by flow cytometry. Representative FACS results of the phagocytosis assay are presented in the left panel. (n = 3).

**
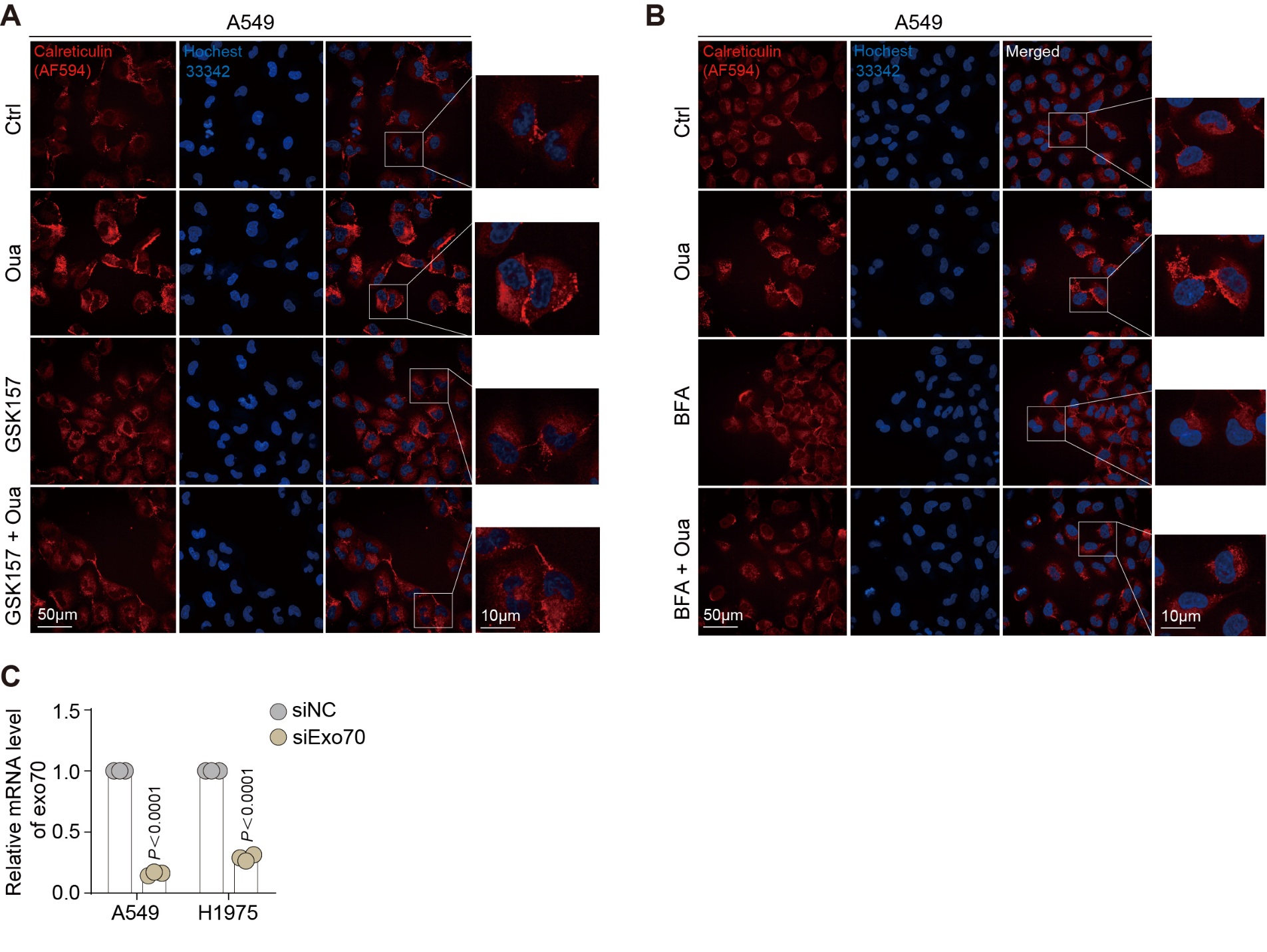
**

**Figure S4. GSK157, BFA and knockdown of exo70 reversed ecto-CRT expression. (A)** GSK157 was used to inhibit PERK activation. After ouabain or digoxin treatment, the ecto-CRT was detected by confocal imaging system. (scale bar: 50 µm and 10 µm) **(B)** Cancer cells were pretreated with BFA for 1 h and removed, followed by treatment with ouabain. The ecto-CRT was detected by confocal imaging system. (scale bar: 50 µm and 10 µm) **(C)** The mRNA level of exo70 was detected by qPCR to explore the knockdown efficacy of siRNA targeting exo70. (n = 3).
